# Computational-Intelligence-Based Scheduling with Edge Computing in Cyber–Physical Production Systems

**DOI:** 10.3390/e25121640

**Published:** 2023-12-09

**Authors:** Changqing Xia, Xi Jin, Chi Xu, Peng Zeng

**Affiliations:** 1 State Key Laboratory of Robotics, Shenyang Institute of Automation, Chinese Academy of Sciences, Shenyang 110016, China; xiachangqing@sia.cn (C.X.); jinxi@sia.cn (X.J.); xuchi@sia.cn (C.X.); 2 Key Laboratory of Networked Control Systems, Chinese Academy of Sciences, Shenyang 110016, China; 3 Institutes for Robotics and Intelligent Manufacturing, Chinese Academy of Sciences, Shenyang 110016, China

**Keywords:** manufacturing, CPPS, edge computing, real-time, scheduling

## Abstract

Real-time performance and reliability are two critical indicators in cyber–physical production systems (CPPS). To meet strict requirements in terms of these indicators, it is necessary to solve complex job-shop scheduling problems (JSPs) and reserve considerable redundant resources for unexpected jobs before production. However, traditional job-shop methods are difficult to apply under dynamic conditions due to the uncertain time cost of transmission and computation. Edge computing offers an efficient solution to this issue. By deploying edge servers around the equipment, smart factories can achieve localized decisions based on computational intelligence (CI) methods offloaded from the cloud. Most works on edge computing have studied task offloading and dispatching scheduling based on CI. However, few of the existing methods can be used for behavior-level control due to the corresponding requirements for ultralow latency (10 ms) and ultrahigh reliability (99.9999% in wireless transmission), especially when unexpected computing jobs arise. Therefore, this paper proposes a dynamic resource prediction scheduling (DRPS) method based on CI to achieve real-time localized behavior-level control. The proposed DRPS method primarily focuses on the schedulability of unexpected computing jobs, and its core ideas are (1) to predict job arrival times based on a backpropagation neural network and (2) to perform real-time migration in the form of human–computer interaction based on the results of resource analysis. An experimental comparison with existing schemes shows that our DRPS method improves the acceptance ratio by 25.9% compared to the earliest deadline first scheme.

## 1. Introduction

The technological evolution of the Industrial Internet of Things (IIoT), human–computer interaction (HCI) [1] and computational intelligence (CI) [2] is providing new solutions for Industry 4.0 to enable the realization of flexible customized production. By deploying sensor nodes and industrial gateways in a factory, the production equipment can be endowed with the ability to perform data collection, protocol conversion and other local operations. Then, the industrial cloud uses CI methods to determine the production schedule and processing parameters based on both the uploaded data and the customization requirements [3]. The dispatching of computing jobs (such as solving control instructions, scheduling algorithms, etc.) can be regarded as a job-shop scheduling problem (JSP), which is a classical NP-hard problem [4]. The purpose of solving a JSP is to guarantee the safe and reliable execution of computing jobs through optimal job scheduling or resource reservation. Unfortunately, due to the requirements for ultralow latency (10 ms) and ultrahigh reliability (99.9999% in wireless transmission) of the control instructions [5], current cloud-based scheduling models and CI methods with random characteristics cannot be effectively applied for operation-level control.

Intelligence sinking based on edge computing technology is an efficient solution to achieve the ultralow latency requirements for operation-level control. Because of its characteristics of being closer to the end equipment than the cloud is and having higher computing power than embedded devices do, edge computing technology can maximize the advantages of HCI and CI to increase the response speed of operation-level control instructions. Currently, most works on edge computing have focused on task offloading under various conditions, e.g., energy-constrained conditions [6], latency-aware conditions [7,8,9], Internet of Vehicles scenarios [10,11] and the convergence of edge computing and AI for UAVs [12]. However, these works have considered only soft real-time scenarios, and the existing methods cannot meet the requirements for both ultralow latency and ultrahigh reliability.

Furthermore, in Industry 4.0, some unexpected critical jobs may arise that must be addressed within a very short time, such as production changes [13], virtual reality or augmented reality in a smart factory [14], a relationship model in a microdrilling process of a sintered tungsten–copper alloy [15] and additional customization requirements (e.g., the upper bound on the latency for virtual reality is 13 ms [16]). The latest research work has begun to consider this issue. In terms of offloading cost: Eshraghi et al. study the problem of the joint offloading decision and resource allocation for mobile cloud networks with a computing access point (CAP) and a remote cloud center to minimize a weighted sum of the average cost and cost variation. Based on this work, [17] focuses on how to select computing tasks to maximize effective rewards in an uncertain and stochastic environment. References [18,19] study distributed task offloading and service management under uncertainty to minimize the overall task computing–communication delay. A distributed digital twins framework to improve decision making at a local level in manufacturing processes is proposed in [20]. In terms of offloading reliability: [21] adopts a two-stage task offloading framework to realize effective server recruitment and reliable task offloading under information asymmetry and uncertainty; then, [22,23,24] consider the optimization of channel selection that is critical for efficient and reliable task delivery. References [25,26] introduce blockchain technology to further improve system security. Reference [27] proposes a URLLC-aware task offloading scheme based on the exponential weight algorithm. Nevertheless, real-time performance and reliability cannot be treated in isolation within industrial production, and only a few existing works establish a deterministic performance boundary. Additionally, there is scarce research focusing on finding a harmonious balance between the uncertainty inherent in intelligent algorithms and the local optimization characteristics of heuristic algorithms.

**Figure 1:**
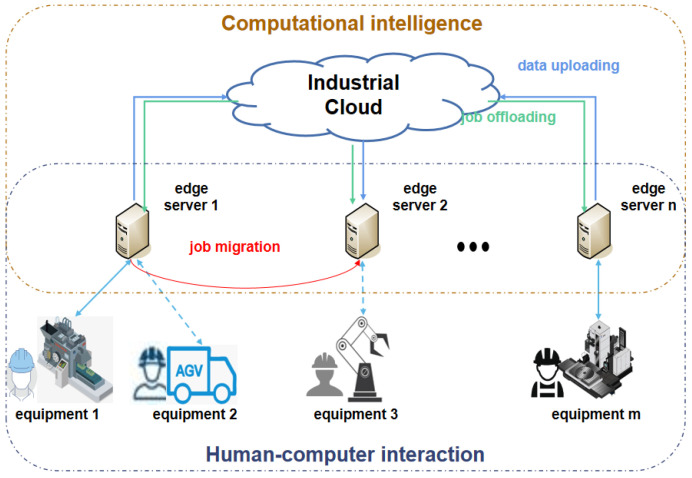
Industrial edge computing system.

Thus, this paper proposes an edge computing system for industrial production scenarios, in which edge servers acting as carriers of jobs offloaded from the cloud are interconnected with the industrial cloud via a wired network, as shown in Figure 1. This system contains a large number of pieces of equipment, each of which is responsible for a certain production operation. One or several pieces of equipment are connected to each edge server, which can collect and preprocess the data and system states of the connected equipment through IIoT devices before uploading this information to the cloud [28,29]. In our design, edge servers possess partial local decision-making capabilities utilizing CI methods, encompassing tasks such as job migration, control instruction generation and job execution. HCI primarily serves the purpose of error correction, maintaining the highest priority in this industrial edge computing system. When unexpected jobs are present in the system, the system can schedule and migrate them using the later-proposed DRPS scheduling algorithm, ensuring real-time job performance and achieving the predetermined system performance indicators.

This paper addresses the challenge of efficiently utilizing the limited computational and communication resources of edge servers to achieve low-latency and high-reliability execution for each job in hard real-time scenarios. By combining real-time scheduling theory with the computational intelligence prediction algorithm (without loss of generality, the classical backpropagation (BP) neural network [30] was chosen as the benchmark prediction algorithm), a dynamic resource prediction scheduling (DRPS) method based on CI is proposed to solve this problem. The core ideas of the proposed DRPS method are (1) to predict job arrival times based on a BP neural network and (2) to perform real-time migration in the form of HCI based on the results of resource analysis and prediction accuracy. A BP neural network is a mainstream CI algorithm that can determine the mapping relationship between certain input factors and the corresponding output based on a historical training sample. By taking advantage of the capabilities of a BP neural network, the proposed DRPS method can predict the arrival times of jobs offloaded from the industrial cloud. Furthermore, when given a certain reliability parameter, DRPS can be used to adjust the scheduling policy based on the results of resource analysis to reduce the uncertainty of prediction and meet the demands of industrial production in the form of HCI.

The main contributions of this paper are listed as follows:

(1) To the best of our understanding, no previous works have studied CI-based prediction for high-reliability real-time scheduling in an industrial system with edge computing capabilities. This paper is the first to propose a job scheduling method that makes limited use of prediction results obtained via CI techniques to solve the JSP in a high real-time and high-reliability cloud–edge collaboration scenario.
(2) To meet the requirements for industrial customized production (especially for unexpected jobs), this paper proposes a DRPS method to establish a trade-off between resource utilization and system performance. That is, DRPS enables the dynamic adjustment of the scheduling policy to meet the industrial requirement based on a given reliability parameter. Furthermore, DRPS permits localized migration of unexpected jobs, thereby mitigating the uncertainty associated with CI techniques and improving the system response speed. The results of both numerical simulations and physical experiments indicate the effectiveness of our method.

The rest of the paper is organized as follows. Section 2 describes and models the problem of dynamic conditions in CPPS. Section 3 provides an explanation of the proposed dynamic prediction scheduling method. The experimental results of the proposed DRPS are analyzed in Section 4, and finally, Section 5 concludes the paper.

## 2. Problem Description

Consider an industrial system based on edge computing, as illustrated in Figure 1. Let the edge server set be N={1,2…n}, and let the number of pieces of equipment be *M*. The characteristics of job *i* in the job set *J* are denoted by $\left\{c_{i},d_{i},t_{i}\right\}$, where the execution time for job *i* is $c_{i}$, the deadline is $d_{i}$ and the period (minimum separation) is $t_{i}$. Similarly, job *k* in the unexpected job set *K* can be represented by $\left\{c_{j},d_{j}\right\}$ (all these parameters are expressed in units of slots).

Considering that the probability of unexpected job occurrence is low, it is assumed that there will be at most one unexpected job in the system within any given time window (the number of unexpected jobs can be easily extended by setting the length of time window). Furthermore, the absolute deadline of *i* can be calculated as $a_{i}+d_{i}$ when the arrival time of job *i* is denoted by $a_{i}$. The objective in this paper is to minimize the impact of unexpected jobs in a hard real-time scenario. That is, when J={1,2…j} regular jobs are offloaded from the cloud and K={1,2,…k} unexpected jobs arise, the objective is to determine how to schedule these (J+K) jobs such that the requirements of the industrial system are met.

Different from the jobs in a traditional JSP, the offloaded job set in this work consists of sporadic tasks but not traditional periodic ones. A job arrives at the assigned edge server when (1) there is production demand, (2) the server has sufficient resources to schedule the job before its deadline and (3) the connected equipment can perform the corresponding operations. Based on these requirements, the job execution time can be bounded. However, the nature of such sporadic tasks results in low resource utilization. The edge server knows that the same job *i* cannot arrive twice within a time interval $t_{i}$, but it does not know the exact arrival time of job *i*. This is the core problem resulting in the inefficient use of edge resources, which also has a great influence on the execution accuracy for unexpected jobs. Hence, to improve the schedulability of the (J+K) jobs, two challenges must be addressed:

(1) Fast response: when unexpected jobs occur, how to achieve real-time selection of an edge server with little or no transmission with the cloud.
(2) Performance guarantee: how to dynamically migrate these unexpected jobs before the system runs out of computational resources, such as when a regular job with high resource utilization arrives on the selected edge server.

Clearly, an event-triggered mechanism is used for scheduling and migrating unexpected jobs to enhance communication resource efficiency. When a sporadic job is detected, it activates the corresponding processing strategy. Given that this work primarily addresses real-time scheduling issues for unexpected jobs, no specific constraints are imposed on the event-trigger method itself. Optimization of the event-trigger method follows approaches proposed in References [31,32] et al.

The schedulability of the job set is defined as follows. The job set is schedulable when all jobs can meet their deadlines; otherwise, the job set is unschedulable. Considering the critical nature of job set *K*, the objective in this work can be formulated as

(1) $$\begin{array}{cc} \; & R>\frac{\left|S\right|}{\left|J|+|K\right|}=R^{*},\\ \; & s.t.\\ \; & \tau_{k}\leq d_{k},\forall k\in K,\\ \; & \min\{\tau_{i}>d_{i},i\in J\}, \end{array}$$

where *R* is the system performance evaluation parameter; $R^{*}$ is the given performance index for the system, which is usually set to 99.99% or 99.9999% in industrial systems; *S* is the scheduled job set; and $\tau_{i}$ is the actual execution time of job *i*. Hence, the system performance *R* can be evaluated using Equation (1). The scheduling algorithm is deemed accurate when the system meets its specified performance index. That is, the job set can simultaneously satisfy its real-time response and reliability requirements when $R>R^{*}$.

## 3. Dynamic Resource Prediction Scheduling

**Figure 2:**
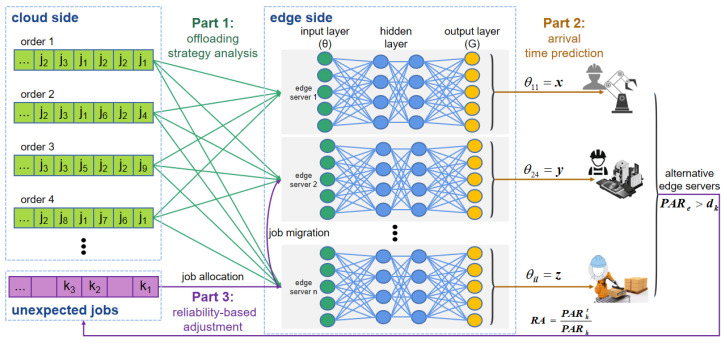
Flowchart of DRPS for an industrial edge computing system.

In this section, a DRPS method is proposed to address the challenges introduced above. As shown in Figure 1, the system consists of three levels: the cloud level, the edge level and the equipment level. First, computing jobs are offloaded from the cloud level to the edge level; then, edge servers schedule the jobs and assign equipment to execute the jobs. DRPS mainly focuses on the work performed at the edge level and can be divided into three steps: offloading strategy analysis, arrival time prediction and reliability-based policy adjustment. A flowchart of DRPS is shown in Figure 2.

### 3.1. Offloading Strategy Analysis

There are many kinds of offloading strategies that can be applied in cloud–edge collaboration scenarios [33]. To simplify the derivation, the earliest deadline first (EDF) scheme is adopted in our design as the baseline job set offloading strategy. The EDF scheme is an optimal scheduling strategy for preemptive uni-processors and is widely used in industrial systems [34]. The cloud adopts the EDF offloading strategy to dispatch jobs depending on their absolute deadlines and the resource utilization of each edge server; specifically, job *i* will be dispatched earlier than job *j* when $d_{i}-a_{i}<d_{j}-a_{j}$, and edge server *k* will receive job *i* when *k* has the lowest utilization among all edge servers that could execute job *i*. The edge server utilization is used in this work to describe the maximum resource utilization when occupied with the regular job workload, which can be calculated as

(2) $$\begin{array}{cc} \; & U_{k}=\sum_{i=1}u_{i},\\ \; & u_{i}=\frac{c_{i}}{T}, \end{array}$$

where $T=LCM\{t_{i},i\in J_{k}^{*}\}$ is the least common multiple of the job periods on edge server *k* and $J_{k}^{*}$ is the historical average number of executable jobs on edge server *k*, $J=\sum_{k=1}^{\left|N\right|}J_{k}^{*}$.

**Figure 3:**
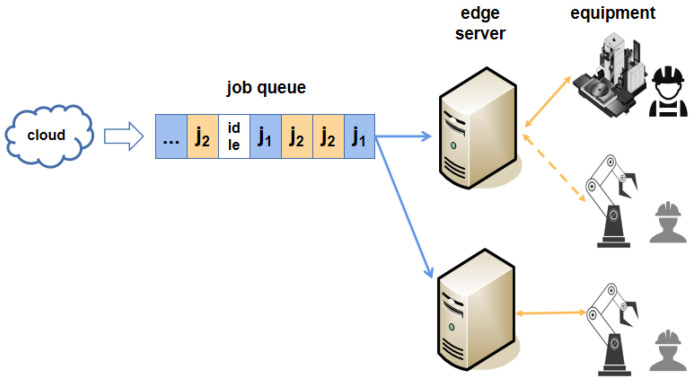
Process of job arrival on an edge server.

For each edge server, the characteristics of the offloaded jobs are partially unknowable. Figure 3 depicts the process of job arrival on an edge server. Two kinds of jobs can be offloaded to the edge server, where $j_{1}=\left\{1,2,2\right\}$ and $j_{2}=\left\{1,1,1\right\}$. Initially (slot 0), only $j_{1}$ is in the queue; then (slots 1–3), as jobs continue to arrive, the edge server executes jobs based on their absolute deadlines. When there is no demand (slot 4), the edge server is idle. Hence, the minimum length of an idle interval on an edge server is $\left[0,\left(\min\left\{t_{i}\right\}-1\right)\right]$ when jobs are arriving periodically; otherwise, the maximum idle interval is positive infinity. To achieve efficient job migration on the edge side, it is necessary to accurately predict the arrival times of jobs on each edge server.

### 3.2. Arrival Time Prediction

An arrival time prediction method (ATPM) based on a BP neural network is proposed to forecast the job arrival times on each edge server. The ATPM network is a multilayered neural network that, for a given state *s*, outputs a vector of idle values G(s,·;β), where β is a parameter of the network that is associated with the job properties. Each time interval θ is calculated as the difference between two consecutive arrival times: $\left\{\theta_{11}=a_{1}-0,\theta_{12}=a_{2}-0\ldots \right\}$. To simplify computations, it is assumed that the transition time on an edge server is negligible, the edge servers and equipment are consistently available and operational under normal conditions, and all edge servers possess the same computational capacity (this can be easily extended to heterogeneous computing servers by adjusting $c_{i}$). One caveat is in order. Considering the computational capacity of each distributed edge server, the ATPM model should first be trained based on the historical workload in the remote or local cloud; then, incremental learning can be achieved with fewer resources based on the most recent job states after deployment.

The flowchart of the ATPM is shown in part 2 of Figure 2. The ATPM network is divided into an input layer, a hidden layer and an output layer. For each instance of the ATPM model, the input layer includes $|J^{*}|+3$ neurons, consisting of $|J^{*}|$ arrival times and three features of the edge server workload. The three features of the edge server workload are listed as follows:

(1) Max(θ): the maximum arrival interval among all jobs in $J^{*}$; in this work, it is assumed that the maximum arrival interval for a job is twice its period.
(2) Min(θ): the minimum arrival interval among all jobs in $J^{*}$; in this work, the minimum arrival interval for a job is set as equal to its period.
(3) |N|: number of edge servers.

Based on these features, the output layer of the ATPM network consists of $J^{*}$ neurons. Each neuron outputs an estimated arrival interval in the next round. The historical data set of job arrival times is denoted by *A*. The learning rate is η. The number of iterations is denoted by α, with $\alpha_{max}$ being the maximum number of iterations. The given permissible error for the system is ϵ. Since the edge servers are connected to each other via an industrial Ethernet protocol, the workload of each server on the edge side in real time can be obtained.

In Algorithm 1, given the job arrival times *A*, the workload features and the learning rate η, the set of jobs to be executed can be obtained for edge server *e* (lines 1–3); then, a neural network can be constructed based on $|J^{*}|+3$ input neurons and $J_{e}^{*}$ output neurons (lines 4–5). The initial weights of the hidden layer are generated randomly and are then adjusted in accordance with the sample outputs and the objective function. After computation through the hidden layer, the ATPM algorithm assesses whether the iterative process needs to continue or stop based on given parameters such as the upper bound on the number of iterations and the termination error (lines 6–12). Finally, the ATPM function set is returned. The minimum value calculated by the ATPM is the arrival time of the next job on edge server *e*.
**Algorithm 1** Arrival time prediction method**Input:** the historical data set of job arrival times *A* and workload features for the current edge server; the learning rate η**Output:** the schedulability of the emergency flow and the acceptance ratio for regular flows  1:function set ATPM(A,Max(θ),Min(θ),|N|,η)  2:**for** each edge server *e* in *N* **do**  3:   obtain $J_{e}^{*}$ and the characteristics of the jobs in $J_{e}^{*}$  4:   **for** all data θ∈A **do**  5:     construct a neural network with $|J_{e}^{*}|+3$ input neurons and $J_{e}^{*}$ output neurons  6:     train the network and update the weights based on gradient descent  7:     **if** $\alpha >\alpha_{max}\cup E\left(\alpha \right)\leq \epsilon$ **then**  8:        **return** $ATPM_{e}\left(A,Max\left(\theta \right),Min\left(\theta \right),|N|,\eta \right)$  9:        break10:     **end if**11:   **end for**12:**end for**13:**return** function set ATPM(A,Max(θ),Min(θ),|N|,η)

For training in the cloud, the target edge server can be regarded as a special edge server that can execute all jobs in *J*; correspondingly, for further training on each individual edge server, incremental learning is adopted to optimize the weights based on the particular features of that edge server.

### 3.3. Reliability-Based Policy Adjustment

In the preceding two subsections, the problems of centralized offloading and distributed prediction were investigated. However, these two methods still cannot solve the hard real-time problem in flexible CPPS, especially when unexpected jobs arise. To address this issue, a DRPS method is proposed in this subsection. The key idea is to analyze the system schedulability within a given reliability by means of real-time system resource analysis [35,36,37].

**Figure 4:**
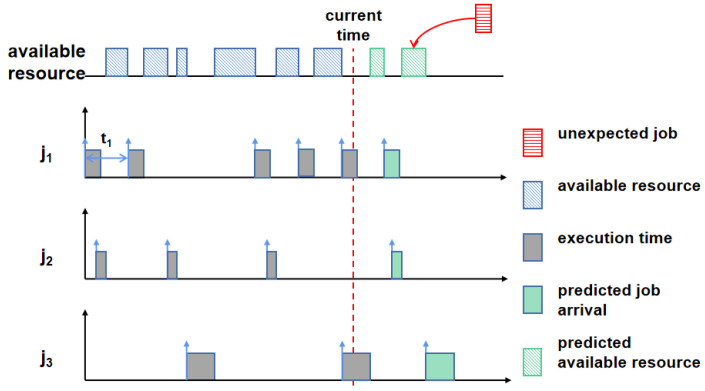
Predicted available resources.

Based on the results from the above two subsections, the bounds on the arrival time interval for job *j* in $J_{e}^{*}$ are $\left[a_{j}+t_{j},a_{j}+\theta_{j}\right)$. Thus, the workload conditions of edge server *e* with three jobs in $J_{e}^{*}$ can be depicted as shown in Figure 4. The workload conditions of edge server *e* are represented to the left of the red line; correspondingly, the predicted conditions are shown in the right part of the figure. Based on the workload conditions, edge server *e* can forecast its future available resources in the next round. An unexpected job *k* can be scheduled if the predicted available resources are no less than the job’s demand.

By the definition of the predicted available resources on edge server *e*, denoted by ${PAR}_{e}$, unexpected job *k* can be scheduled on *e* when $c_{i}\leq PAR_{e}\leq d_{k}$; otherwise, job *k* needs to be migrated to another edge server. Hence, the DRPS method consists of the following two mechanisms.

(1) CI-based first-fit offloading: When the predictive accuracy of the ATPM is no less than the given reliability index *R*, unexpected job *k* first searches for an edge server *e* with sufficient available resources, $c_{k}\leq PAR_{e}$; if none of the edge servers meet this condition, job *k* is assigned to the first edge server on which resources become available.
  When the predictive accuracy of the ATPM is less than *R*, job *k* is directly assigned to the first edge server on which resources become available.
(2) HCI-based high-accessibility migration: When insufficient resources are available to complete job *k*, job *k* is immediately migrated when its execution on the current edge server is suspended. The migration target is chosen as the edge server *h* with the highest resource accessibility *RA*, which can be calculated as
  (3) $$RA=\frac{PAR_{h}^{t}}{PAR_{h}},$$
  where $PAR_{h}^{t}$ represents the ascertainable resources calculated based on the characteristics of the sporadic jobs.

Based on these two mechanisms, DRPS can enable multiscale adjustments across multiple edge servers. Furthermore, all analysis and decision-making processes for DRPS are performed locally, thereby eliminating the time overhead incurred in networked control systems.

## 4. Experimental Results

This section evaluates the performance of the proposed DRPS method in comparison with the traditional BP method and the classical EDF policy. Because no previous works have studied predictive real-time scheduling in an industrial system with edge computing capabilities, the experiments focus on two aspects: the comparison of the acceptance ratio of the proposed method with those of the BP and EDF methods using different parameter settings and the construction of a simple real-time control system to evaluate the performance of DRPS under different workloads. The acceptance ratio considered in this section is defined as the percentage of jobs for which both the real-time response and reliability requirements are met.

### 4.1. Simulations

The simulations presented in this work refer to a benchmark JSP data set from [38], from which the instances entitled “Applegate and Cook” [4] and “Lawrence” [39], with different numbers of pieces of equipment and regular jobs, are used. The number of edge servers is randomly generated, and equipment is connected to each edge server to establish the industrial edge computing system. Considering the computational capacity, the number of pieces of equipment connected to each edge server is 1∼5. The chosen network structure is 13×10×1, and the training method employed for the BP neural network is dynamic gradient descent. The training of the entire model is conducted in Matlab, calling upon the toolbox of its neural network.

The parameters in this work are divided into three groups: (1) the parameters of the industrial edge computing system: the number of edge servers is 5∼30, the arrival intervals for each job follow a Poisson distribution and are bounded by 1∼2 times the period, the acceptable system reliability index is generated randomly in the range 0.9∼1, and for each edge server, $J^{*}\in \left[1,5\right]$; (2) the parameters of the BP neural network: the number of neurons in the hidden layer of the BP neural network is $\frac{input+output}{2}$, the upper bound on the number of iterations is ${ℵ}_{max}=5000$, the given permissible error for the system is ϵ=0.9, and the learning rate is η=0.01; and (3) the parameters of the unexpected jobs: the maximum number of simultaneous unexpected jobs is 1, and to allow for transmission delays, the characteristics of each job in *K* are generated following the principle that $c_{k}<d_{K}+1$. The acceptance ratio is employed to assess the performance of each method [40]; the system returns 1 when all jobs in the system can be scheduled, whereas otherwise, it returns 0. Each point in one test is calculated as the average value of the scheduling results under the same industrial edge computing system. By continuously generating unexpected jobs in the industrial system, objective comparison results can be obtained.

In our simulations, the resource utilization is used to control the workload of the entire system. The UUniFast algorithm is used to generate each job’s utilization $u_{i}$ to make the job set more available. The results generated by the UUniFast algorithm follow a uniform distribution and are neither pessimistic nor optimistic for the analysis [41]. Based on the results of the UUniFast algorithm, the deadline of each regular job can be bounded based on its execution time and period. In addition, the system utilization is the sum of the utilization of each regular job.

**Figure 5:**
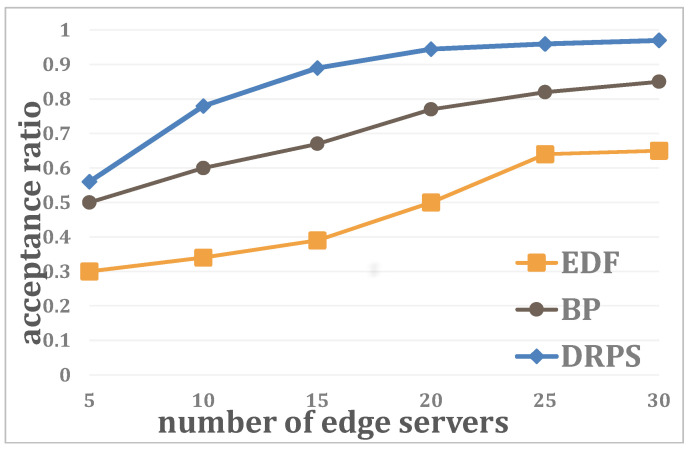
Relationship between the acceptance ratio and the number of edge servers.

First, the relationship between the acceptance ratio and the number of edge servers is analyzed when the number of regular jobs is J=100, the number of unexpected jobs is K=10 and the system utilization is U=0.2. As Figure 5 shows, for all three methods, the performance improves with increasing *N*. Furthermore, DRPS shows the fastest rate of performance growth and remains superior at all times. The reason for this phenomenon is that as the number of edge servers grows, the workload remains fixed but the total available resources, $\sum_{e=1}^{\left|N\right|}PAR$, increase; since DRPS can utilize small amounts of resources by assigning an edge server to perform part of a job and then migrating the remainder of the job to another edge server, DRPS achieves higher utilization than the other two methods. In this scenario, the acceptance ratio of DRPS reaches 90% with approximately 16∼17 edge servers.

**Figure 6:**
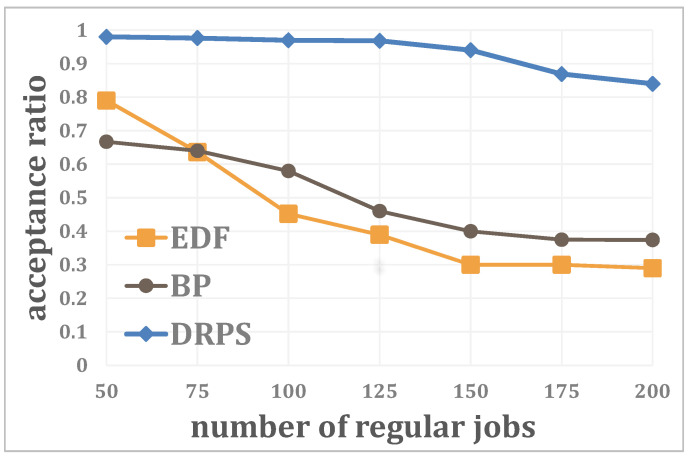
Relationship between the acceptance ratio and the number of regular jobs.

Second, the relationship between the acceptance ratio and the number of regular jobs is analyzed when the number of edge servers is N=15, the number of unexpected jobs is K=10 and the system utilization is U=0.2. Figure 6 shows that DRPS shows the best stability, with its acceptance ratio remaining above 80% at all times. This is due to the ability to efficiently use scattered resources via DRPS. In addition, the performance ranking of the EDF and BP methods reverses when the number of regular jobs reaches 75. This is because the system resource utilization is initially low, meaning that it is easier to select a resource-rich edge server for an unexpected job based on Equation (2) than to predict the future workload; however, as the number of regular jobs increases, the amount of idle resources on each edge server is reduced, causing the performance of the BP method to exceed that of the EDF scheme when the number of regular jobs in the system is greater than 75.

**Figure 7:**
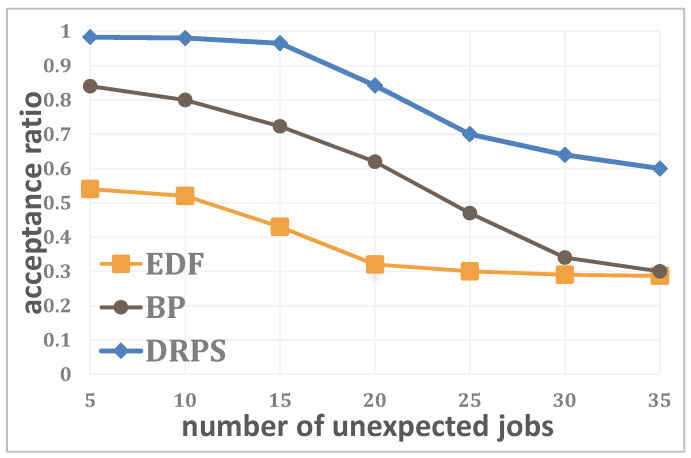
Relationship between the acceptance ratio and the number of unexpected jobs.

Figure 7 shows the relationship between the acceptance ratio and the number of unexpected jobs when the number of edge servers is N=20, the number of regular jobs is J=100 and the system utilization is U=0.2. As expected, for all three methods, the performance decreases with an increasing number of unexpected jobs. Moreover, the performance results always satisfy DRPS>BP>EDF in this scenario because the coupling relationship between an unexpected job and the edge server to which it is offloaded in the EDF and BP methods restricts the ability of these methods to achieve better performance, whereas the use of high-accessibility migration in DRPS improves the system acceptance ratio by allowing better utilization of scattered resources. Even when the number of unexpected jobs exceeds 20, DRPS still maintains a high acceptance ratio (above 80%).

**Figure 8:**
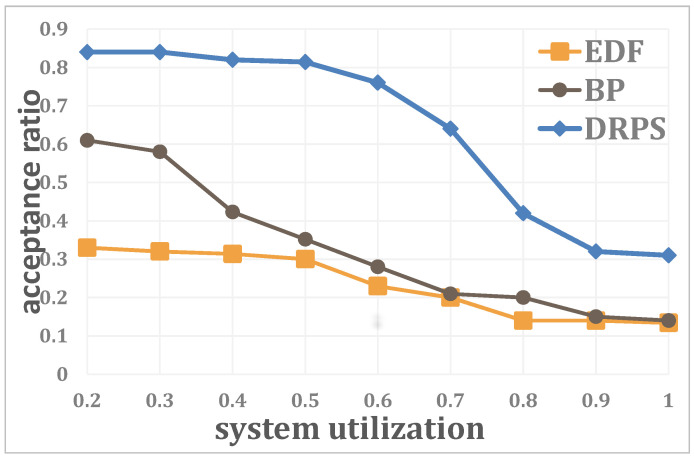
Relationship between the acceptance ratio and the system utilization.

The relationship between the acceptance ratio and the system utilization is shown in Figure 8, where the number of edge servers is N=20, the number of regular jobs is J=100 and the number of unexpected jobs is K=20. For all three methods, the performance decreases with increasing system utilization because the available resources for an unexpected job are reduced. Initially (0.2∼0.6), the downward curve is smoother and slower for DRPS than for the other two methods due to the resource-analysis-based migration mechanism; however, as the utilization continues to increase (0.6∼0.9), the performance of DRPS sharply decreases and then remains at a low level. Nevertheless, the performance of DRPS at a system utilization of 1 is still higher than the performance of the other two methods.

**Figure 9:**
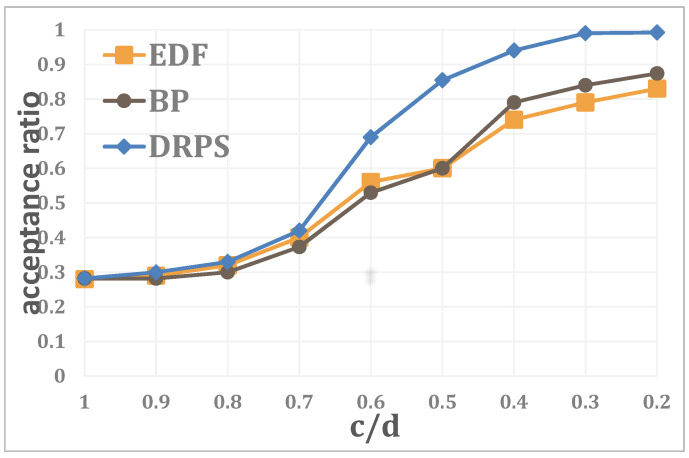
Relationship between the acceptance ratio and the proportion $\frac{c_{k}}{d_{k}}$, k∈K.

In what follows, the relationship between the acceptance ratio and the proportion $\frac{c_{k}}{d_{k}}$, k∈K is analyzed when the number of edge servers is N=20, the number of regular jobs is J=100, the system utilization is U=0.2 and the number of unexpected jobs is K=10. As Figure 9 illustrates, the performance rises rapidly for all three methods as $\frac{c_{k}}{d_{k}}$ increases. This is because as the unexpected jobs become less time sensitive, the system has more opportunities to adjust. Furthermore, the acceptance ratio of DRPS reaches nearly 100% at $\frac{c_{k}}{d_{k}}=0.2$.

**Figure 10:**
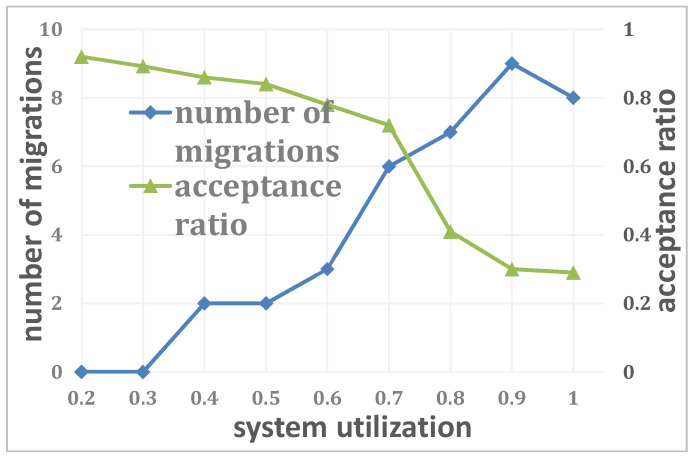
Relationship between the number of migrations and the system utilization.

To evaluate the migration mechanism of DRPS, Figure 10 shows the relationship between the number of migrations and the system utilization when the number of edge servers is N=20, the number of regular jobs is J=100, $\frac{c_{k}}{d_{k}}\leq 0.6$ (k∈K) and the number of unexpected jobs is K=20. A job is migrated when its execution cannot be completed on the current edge server. Hence, no migration is necessary when the system has sufficient resources to execute an unexpected job, and with increasing *U*, DRPS can maintain its performance by continually migrating the job; however, this migration mechanism cannot completely solve the problem under all conditions. Job migration stops when job *k* misses its deadline, that is, $PAR>d_{k}$.

### 4.2. Experiments

**Figure 11:**
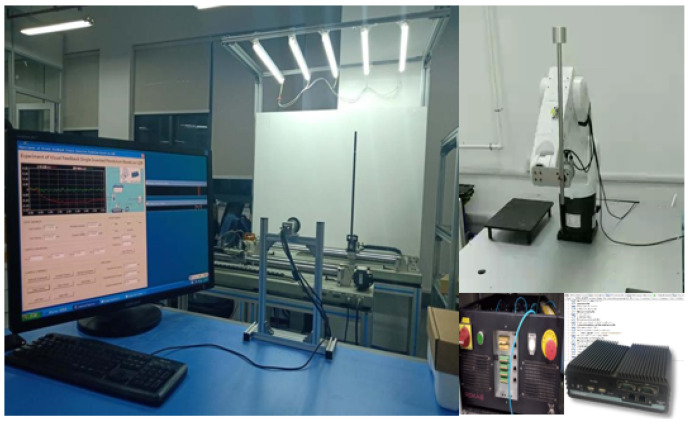
Our real testbed.

A simple verification platform has been established by us, as depicted in Figure 11. In the design, the problem of solving the inverted pendulum angle is taken as the basis for the unexpected job set, with a sampling frequency of 10 ms for the swing angle. That is, the DRPS algorithm needs to select an edge server to calculate the velocity and angular acceleration of the pendulum rod, migrate the job when the resources are insufficient and send the result to a servo motor for a real-time response within 10 ms.

Limited by computational capacity, the characteristics of each unexpected job are set to $c_{k}=2$ ms and $d_{k}=5$ ms. The rules for generating regular jobs align with those used in the simulations. The system has three edge servers, and the number of regular jobs is set to J=20. System utilizations of 0.2, 0.3, 0.5 and 0.7 are considered. Following the downloading of the trained model to each edge server, the performance of DRPS can be assessed by observing the posture of the inverted pendulum.

**Figure 12:**
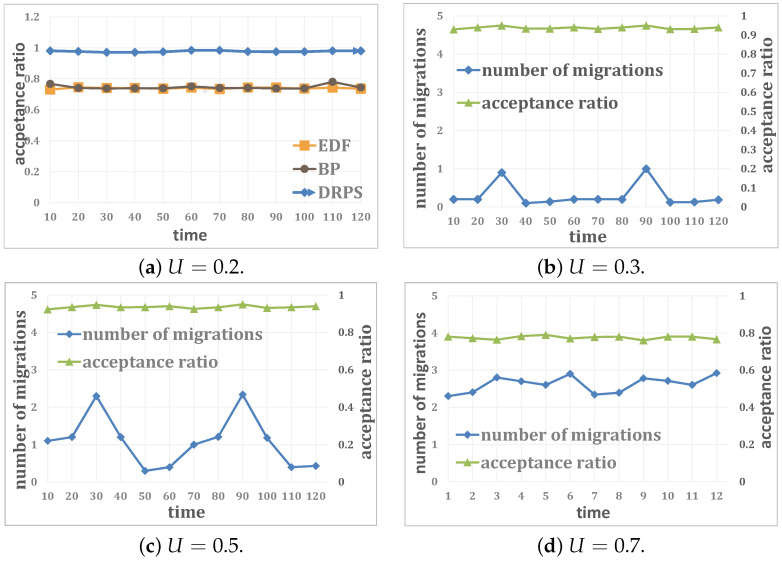
System performance.

The performance of DRPS in our testbed is shown in Figure 12, where the x-axis represents our test time (120 s) and each point in Figure 12 is the average acceptance ratio or number of migrations over 10 s under DRPS. In Figure 12a, the acceptance ratio under DRPS always remains above 97%, representing a performance improvement of 25.9% compared to the EDF scheme and the original BP algorithm. This also indicates that A can to some extent balance the uncertainty of intelligent algorithms and the local optimization nature of heuristic algorithms. The other results in Figure 12 show that DRPS can achieve an acceptance ratio of almost 80% when the system utilization is no higher than 0.7; this performance can meet the requirements for many soft real-time scenarios. Furthermore, the acceptance ratio of DRPS always remains above 90% when the system is operating under low-workload conditions, and the inverted pendulum remains upright at all times. Figure 12 illustrates that the system performance can be substantially improved by reducing the system utilization. Moreover, the system performance can be further enhanced under DRPS by means of selective migration based on resource analysis.

## 5. Conclusions

In conclusion, this paper introduces a computational-intelligence-based dynamic resource prediction scheduling (DRPS) method for efficient utilization of limited computational and communication resources in edge servers, aimed at achieving low-latency and high-reliability execution in hard real-time scenarios within CPPS. The DRPS method employs two core mechanisms: (1) CI-based first-fit offloading and (2) HCI-based high-accessibility migration. The first-fit mechanism utilizes coarse-grained offloading for unexpected jobs, leveraging the prediction results of a BP neural network. The selection of edge servers in the first-fit mechanism is based on two judgment criteria: $c_{k}\leq PAR_{e}$ and the arrival time of available resources. Meanwhile, the high-accessibility migration mechanism ensures fine-grained migration to prevent job deadline misses resulting from inaccurate predictions. To the best of our knowledge, this work is the first to propose a job scheduling method that minimally utilizes prediction results to address the job scheduling problem (JSP) in a cloud–edge collaboration scenario. The experimental results demonstrate that our method enhances schedulability, leading to a 25.9% improvement in the acceptance ratio compared to the EDF scheme.

Looking ahead, future research will be extended to incorporate CI-based prediction for unexpected jobs. Accurate prediction of arrival times for both regular and unexpected jobs can eliminate the indeterminacy in flexible customized production, allowing the application of classical JSP methods. Additionally, conducting an in-depth study of training parameters, such as the optimal number of hidden layers and neurons per hidden layer, would provide valuable insights. Finally, the direct application of DRPS in hard real-time industrial scenarios faces several limitations, including system utilization, the number of unexpected jobs, the number of edge servers and the type of edge server platform. While increasing the number/type of edge servers can address some challenges, others pose significant obstacles to DRPS in real industrial scenarios. Hence, urgent efforts are needed to explore solutions to overcome or circumvent these hindrances.

## Data Availability

Data are contained within the article.
